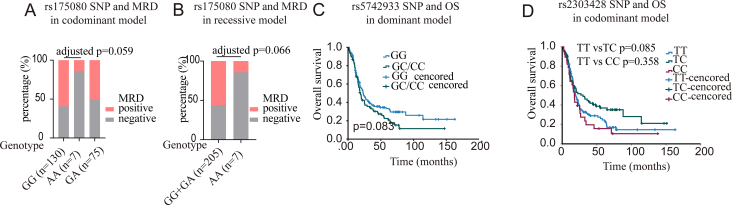# Polymorphisms of mismatch repair pathway genes predict clinical outcomes in acute myeloid leukemia patients

**DOI:** 10.1016/j.gendis.2025.101774

**Published:** 2025-07-16

**Authors:** Amin Zhang, Wancheng Liu, Can Can, Xiaodong Guo, Hexiao Jia, Yihong Wei, Hanyang Wu, Chunyan Ji, Daoxin Ma

**Affiliations:** a Department of Hematology, Qilu Hospital of Shandong University, Jinan, Shandong 250012, China; b Department of Pediatrics, Qilu Hospital of Shandong University, Jinan, Shandong 250012, China

Acute myeloid leukemia (AML) is an aggressive hematological malignancy with a poor prognosis. Cytarabine (Ara-C), a cornerstone of AML chemotherapy, causes DNA damage.^1^ However, patient AML blasts can develop Ara-C resistance. Therefore, there is an urgent need to explore new targets for the treatment of AML. DNA mismatch repair (MMR) pathway genes significantly contribute to the repair process by identifying DNA damage.^2^ The MMR system includes several MMR proteins, such as mutL homolog 1 (MLH1), MLH3, mutS homolog 2 (MSH2), MSH3, MSH6, postmeiotic segregation increased 1 (PMS1), and PMS2. Genetic variations in MMR genes affect individuals' ability to repair chemotherapeutic agent-induced DNA damage.^3^ For instance, *MLH1* rs1799977 AG/GG genotype displayed an increased death risk in diffuse large B-cell lymphoma.^4^ The GG genotype of *MSH2* rs3732183 is correlated with lower recurrence risk, and the GG genotype of *MLH1* rs1800734 carriers is linked with higher overall survival (OS) in oral squamous cell carcinoma.^5^ However, research on the prognostic relationship between MMR and AML post-chemotherapy is lacking, leading us to investigate the polymorphism of MMR pathway genes and their prognostic significance in AML.

We used the MassARRAY platform for genotyping. Briefly, this system involves a multiplex PCR and is followed by MALDI-TOF mass spectrometry analysis. Detailed methods regarding the MALDI-TOF mass spectrometry analysis are presented in the supplementary materials.

Nine single-nucleotide polymorphisms (SNPs) of MMR pathway genes were detailed in Table S1. *MSH3* rs26279 and *MSH6* rs1042821 were rejected because their Hardy–Weinberg equilibrium was less than 0.05. Demographic and clinical characteristics of AML patients were summarized in Table S2. We investigated the association between SNPs and clinical features of AML. In AML, high white blood cell (WBC) level, defined as a WBC count greater than 100 × 10^9^/L, is linked to a poor prognosis. Preliminary chi-square test screening indicated a significant correlation between *PMS1* rs5742933 and WBC count under the codominant model (*p* = 0.039) and dominant model (*p* = 0.012) (Table S3). Univariate logistic regression analysis indicated that in the codominant model, *PMS1* rs5742933 GC and GC/GG genotypes were significantly associated with high WBC level (GC: OR = 3.36, 95% CI = 1.27–8.91, *p* = 0.015; GC/GG: OR = 3.21, 95% CI = 1.23–8.35, *p* = 0.017) (Fig. 1A, B). Additionally, a platelet count below 20 × 10^9^/L was classified as a low platelet level. Preliminary chi-square test screening indicated that the GA/AA genotype of *MLH3* rs175080 was associated with a higher platelet level (*p* = 0.03; Table S4). Univariate logistic regression analysis indicated that the GA/AA genotype of *MLH3* rs175080 was associated with a decreased risk of lower platelet level in the dominant model (OR = 0.44, 95% CI = 0.212–0.914, *p* = 0.028; Fig. 1C).

**Figure 1:**
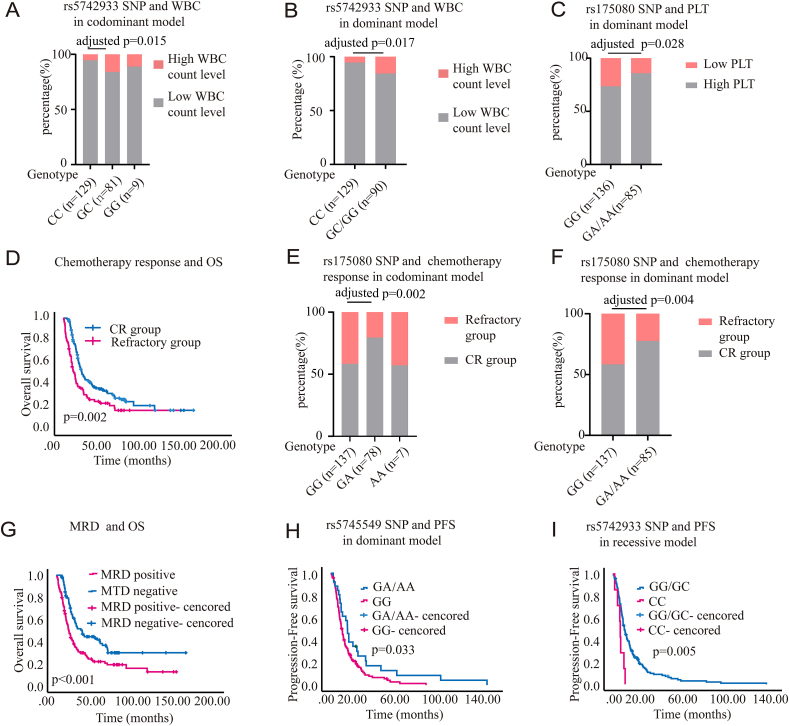
The association of single-nucleotide polymorphisms (SNPs) with clinical outcomes in acute myeloid leukemia (AML) patients. **(A)***PMS1* rs5742933 and white blood cell (WBC) count in the codominant model. **(B)***PMS1* rs5742933 and WBC count in the dominant model. **(C)***MLH3* rs175080 and platelet (PLT) count in the dominant model. **(D)** The Kaplan–Meier curves showing the association of chemotherapy response with overall survival (OS) in AML patients. Complete remission (CR) group: *n* = 144; refractory group: *n* = 76. **(E)***MLH3* rs175080 SNP and chemotherapy response in the codominant model. **(F)***MLH3* rs175080 SNP and chemotherapy response in the dominant model. **(G)** The Kaplan–Meier curves showing the association of minimal residual disease (MRD) group with OS in AML patients. MRD-negative group: *n* = 96; MRD-positive group: *n* = 114. *p* < 0.001. **(H)***MSH4* rs5745549 and progression-free survival (PFS) in the dominant model (GA/AA *vs.* GG, adjusted *p* = 0.033). **(I)***PMS1* rs5742933 and PFS in the recessive model. *p* values were adjusted for sex and age.

We further assessed the sensitivity of induction chemotherapy in non-M3 AML patients. After administering full-intensity induction chemotherapy to 222 AML patients, 76 cases failed to reach complete remission even after two cycles, which were classified as the “refractory” group. Survival analysis showed a significant difference in the median OS between the complete remission and refractory groups (54.7 months *vs.* 39.3 months, *p* = 0.002; Fig. 1D). After preliminary chi-square test screening, the GA in *MLH3* rs175080 in the codominant model and GA/AA in the dominant model were significantly correlated with induction chemotherapy (*p* < 0.05; Table S5). Univariate logistic regression analysis indicated that after adjusting for age and sex, the GA and GA/AA had a decreased risk in the refractory group (GA: *p* = 0.002, OR = 0.36, 95% CI = 0.18–0.68; GA/AA: *p* = 0.004, OR = 0.40, 95% CI = 0.21–0.74) (Fig. 1E, F). In addition, monitoring minimal residual disease (MRD) is crucial in determining the prognosis and treatment strategy for AML patients. A Kaplan–Meier analysis of 210 patients showed significant differences in outcomes between the MRD-negative and MRD-positive groups (median OS: 67.6 *vs.* 39.3 months; *p* < 0.001; Fig. 1G). Consequently, we investigated the association between SNP and MRD status. The codominant and recessive model of *MLH3* rs170580 demonstrated a moderately significant connection with MRD status after an initial chi-square test screening (*p* = 0.045 and *p* = 0.048, respectively; Table S6). However, the univariate logistic regression analysis revealed that after adjusting for age and sex, the AA genotype of *MLH3* rs170580 showed a nearly significant difference (*p* = 0.059 and *p* = 0.066, respectively; OR < 1; Fig. S1A, B). This indicated that the AA genotype of *MLH3* rs170580 might be linked to a better prognosis in AML.

Subsequently, we assessed the prognostic significance of selected SNPs using Kaplan–Meier analysis. Kaplan–Meier analysis revealed that in the dominant model, the *PMS1* rs5724933 polymorphism caused a borderline significant difference in OS in AML patients (*p* = 0.083; Fig. S1C). In the codominant model, the TC genotype of *MSH2* rs2303428 polymorphism showed a difference approaching statistical significance in OS of AML patients (*p* = 0.085; Fig. S1D). In the multivariable analysis, only *MSH2* rs2303428 was identified as an independent risk factor for OS in the recessive model, while no other SNPs were found to be associated with OS (*p* = 0.027; Table S7). Progression-free survival (PFS) information was available for 180 of the 222 AML patients. In the dominant model, the GA/AA genotype of *MSH4* rs5745549 was significantly associated with longer PFS in AML patients (*p* = 0.033; Fig. 1H). In the multivariable analysis, rs5745549 did not remain an independent predictor for PFS in the dominant model (*p* = 0.091; Table S8). In the recessive model, the CC genotype of *PMS1* rs5742933 was significantly associated with poorer PFS in all patients (*p* = 0.005; Fig. 1I). In multivariable cox analysis adjusting for age, risk group, sex, and WBC count, recessive model of rs5742933 remained as an independent predictor for PFS (*p* = 0.006; Table S9).

We also acquire allele frequency data from the NCBI Allele Frequency Aggregator (ALFA) project, which is integrated and given based on data from the Database of Genotypes and Phenotypes (dbGaP). AML patients exhibited significantly different frequencies of variant gene C of *PMS1* rs5742933 and variant gene A of *MSH4* rs5745549 compared with the East Asian population (*p* < 0.01; Table S10).

In conclusion, we study genetic variations in the MMR pathway in AML patients. We found that *PMS1* rs5742933 was deemed the most clinically impactful due to its association with high WBC count and PFS in AML patients. *MLH3* rs175080 was correlated with MRD positivity and the refractory group of AML. *MSH2* rs2303428 was identified as an independent risk factor for OS. Our findings offer new insights into the therapeutic targets and prognosis in AML patients. We still lack validation from other databases, which remains an important area for future improvement.

## Data availability

The data that support the findings of this study are available from the corresponding author upon reasonable request.

## CRediT authorship contribution statement

**Amin Zhang:** Writing – original draft, Conceptualization. **Wancheng Liu:** Methodology. **Can Can:** Formal analysis. **Xiaodong Guo:** Methodology. **Hexiao Jia:** Software. **Yihong Wei:** Investigation. **Hanyang Wu:** Supervision. **Chunyan Ji:** Writing – review & editing. **Daoxin Ma:** Data curation.

## Ethics declaration

The study was approved by the Institutional Ethics Committee of Qilu Hospital, Shandong University (approval code: KYLL-202204-059). Consistent with the Declaration of Helsinki, informed consent was obtained from all participants.

## Funding

This work was supported by grants from the Natural Science Foundation of Shandong Province (Youth Program, ZR2023QH249) and 10.13039/501100001809National Natural Science Foundation of China (No. 82370173, 32241005, 81873439).

## Conflict of interests

The authors declared no competing interests.

## Figures and Tables

**figs1:**