# Supplementary material for: Polymorphisms of mismatch repair pathway genes predict clinical outcomes in acute myeloid leukemia patients
Source: Genes Dis. 2025 Jul 16;13(1):101774. doi: 10.1016/j.gendis.2025.101774 (PMC12624588; doi:10.1016/j.gendis.2025.101774)
Supplement: Multimedia component 1 [file mmc1.docx]

**Materials and methods**

**Patients and controls**

From January 2010 to January 2023, we enrolled 222 patients with either initial diagnosis or recurrent AML treated with Ara-C from QiLu Hospital of Shandong University, China. The diagnosis of enrolled AML patients was based on National Comprehensive Cancer Network Guidelines for AML. The diagnosis of enrolled AML patients was based on National Comprehensive Cancer Network (NCCN) Guidelines for AML. The exclusion criteria were as follows: incomplete data, age < 14 years, CR stage, immune system diseases, and abnormal mental functions. Healthy control group matched with the case group as for gender and age. The study was approved by the Institutional Ethics Committee of Qilu Hospital, Shandong University (approval code: KYLL-202204-059). Consistent with the Declaration of Helsinki, informed consent was obtained from all participants.

**DNA extraction and SNP genotyping**

Genomic DNA was extracted from bone marrow mononuclear cells (BMMCs) or peripheral blood leukocytes, using the TIANamp blood DNA kit (Tian Gen, China). DNA concentration and purity were assessed using a DeNovix DS-11 Spectrophotometer (DeNovix Inc., Wilmington, DE, USA). After extraction, DNA was stored at −80 °C before being used for genotyping. (1) SNP Sequence Organization: We obtained SNP data from the UCSC genome database and the corresponding reference SNP ID number was obtained through the dbSNP database. Utilizing the dbSNP database, a total of 200 bp of gDNA sequence, including the target SNP sites, were compiled. The UCSC database was then used to verify the genomic homology of these sequences within the gene to assess potential risks associated with genotyping. For each SNP site, three primers were synthesized through PAGE purification: two PCR primers and one UEP primer. All primers were purified by polyacrylamide gel electrophoresis. (2) PCR Amplification Reaction: This reaction amplifies the gene fragment containing the SNP site from the gDNA genome using PCR, yielding a product length of 100–200 bp. A PCR master mix was prepared in a 1.5 mL EP tube, then gently vortexed and briefly centrifuged. Using an 8- or 12-channel pipette, 4 μL of PCR master mix was added to each sample well in a 384-well plate, followed by 1 μL of template DNA (20 ng/μL). The plate was carefully sealed with a 384-well cover film to prevent evaporation during PCR. After centrifuging at 1000 rpm for 1 minute, the plate was placed in a PCR instrument to initiate the amplification according to the specified program. (3) Alkaline Phosphatase Treatment: Following PCR, free dNTPs in the reaction system were phosphorylated by treating the PCR product with SAP (shrimp alkaline phosphatase). This step removes residual dNTPs from the reaction mixture. (4) Single Base Extension Reaction: Using the UEP primer, a single-base extension reaction was performed in a ddNTP system, generating an extension product complementary to the SNP genotype being detected. Uniformly fill the resin in a 384/6MG Dimple plate and allow it to dry for 10 minutes. Add 16 μL of water to each well of the 384 samples plate. Carefully invert the 384 sample plate onto the Dimple plate, tap gently to transfer the resin into each well. Place the 384 sample plate in an inverted centrifuge at room temperature and mix by rotation for 30 minutes. (4) Chip Spotting: Using the MassARRAY Nanodispenser RS1000, transfer the purified extension products onto a 384-well SpectroCHIP bioarray. Analysis with MALDI-TOF Mass Spectromet.

**Statistical analysis**

Statistical analyses were conducted using SPSS version 26.0. SNPs with a P value greater than 0.05 in Hardy–Weinberg equilibrium (HWE) within the general population were included in this study. Genotyping data were analyzed using four models: dominant, recessive, codominant, and allelic frequency models. For preliminary screening, we employed either Fisher's exact test or chi-square test to examine the correlations between SNPs and clinical indicators. Univariate logistic regression was utilized to determine P values adjusted for age and gender, odds ratios (ORs), and 95% confidence intervals (CIs). The Kaplan-Meier method and log-rank tests were employed for survival analysis. Multivariate hazard ratios (HRs) were calculated using Cox proportional hazards regression analysis. The overall survival (OS) was assessed from the time of diagnosis to the time of patient death, regardless of the cause of death. progression-free survival (PFS) was defined as the time period between the initial diagnosis and the first disease progression.

Figure S1 Association of single-nucleotide polymorphisms (SNPs) with clinical outcomes in acute myeloid leukemia (AML) patients. (A) *MLH3* rs175080 and minimal residual disease (MRD) in the codominant model (GG *vs.* AA after adjusted for age and sex, *P* = 0.059). (B) *MLH3* rs175080 and MRD in the recessive model (GG/GA *vs.* AA, adjusted *P* = 0.066). (C) *PMS1* rs5742933 and overall survival (OS) in the dominant model (GC/CC *vs.* GG, *P* = 0.083). (D) *MSH2* rs2303428 and OS in the codominant model (TT *vs.* TC, *P* = 0.085).
