# Supplementary material for: Polymorphisms of mismatch repair pathway genes predict clinical outcomes in acute myeloid leukemia patients
Source: Genes Dis. 2025 Jul 16;13(1):101774. doi: 10.1016/j.gendis.2025.101774 (PMC12624588; doi:10.1016/j.gendis.2025.101774)
Supplement: Multimedia component 2 [file mmc2.docx]

Table S1. Selected genes and SNPs

| Genes | SNPs | Variant | Variant allele | HWE  *P-*value |
| --- | --- | --- | --- | --- |
| MLH1 | rs1799977 | A > G | G | 0.51 |
| MLH3 | rs175080 | G>A | A | 0.31 |
| PMS1 | rs5742933 | G＞C | C | 0.39 |
| MSH2 | rs2303428 | T＞C | C | 0.06 |
| MSH3 | rs26279 | A＞G | G | 0.009 |
| MSH4 | rs5745325 | G>A | A | 0.67 |
| MSH4 | Rs5745549 | G>A | A | 0.52 |
| MSH5 | rs707938 | A＞G | G | 0.21 |
| MSH6 | rs1042821 | G>A | A | 0.04 |

Table S2. The demographic and clinical characteristics of AML patients and controls

| Variable | Case *n* (%) |
| --- | --- |
| Gender *n* (%) |  |
| Male | 113(50.9) |
| Female | 109(49.1) |
| Age (years), median (range) | 51.5(14-79) |
| WBC |  |
| Median (×109/l) *n* (%) |  |
| < 100 × 109/l | 200(90.1) |
| ≥ 100 × 109/l | 22(9.9) |
| PLT |  |
| Median(×109/l) *n* (%) |  |
| > 20× 109/l | 173(78.3) |
| ≤ 20 × 109/l | 48(21.7) |
| Risk stratification *n* (%) |  |
| Favorable | 64(28.8) |
| Intermediate | 127(57.3) |
| Adverse | 31(13.9) |
| Response *n* (%) |  |
| CR group | 151(68.1) |
| Refractory group | 71(31.9) |
| MRD *n* (%) |  |
| MRD positive | 116(54.7) |
| MRD negative | 96(45.3) |

Table S3. Association between WBC count and SNPs in AML

| Genes | SNPs | Genotype | Allele | Low WBC  *n* (%) | High WBC  *n* (%) | Model / allele | Uncorrected *P* value |
| --- | --- | --- | --- | --- | --- | --- | --- |
| MLH1 | rs1799977 | AA |  | 183(91.5) | 19(86.4) | Codominant | 0.428 |
|  |  | AG |  | 17(8.5) | 3(13.6) | Dominant | 0.428 |
|  |  | GG |  | 0 | 0 | Recessive | -- |
|  |  |  | A | 383(95.8) | 41(93.2) | Allele | 0.435 |
|  |  |  | G | 17(4.2) | 3(6.8) |  |  |
| MLH3 | rs175080 | GG |  | 122(61.0) | 15(68.2) | Codominant | 0.689 |
|  |  | GA |  | 72(36.0) | 6(27.3) | Dominant | 0.511 |
|  |  | AA |  | 6(3.0) | 1(4.5) | Recessive | 0.523 |
|  |  |  | G | 316(79) | 36(81.8) | Allele | 0.662 |
|  |  |  | A | 84(21) | 8(18.2) |  |  |
| PMS1 | rs**5742933** | GG |  | 122(61.6) | 7(33.3) | Codominant | **0.039** |
|  |  | GC |  | 68(34.3) | 13(61.9) | Dominant | **0.012** |
|  |  | CC |  | 8(4.1) | 1(4.8) | Recessive | 0.604 |
|  |  |  | G | 312(78.8) | 27(64.3) | Allele | **0.033** |
|  |  |  | C | 84(21.2) | 15(35.7) |  |  |
| MSH2 | rs2303428 | TT |  | 94(47.7) | 12(54.5) | Codominant | 0.792 |
|  |  | TC |  | 77(39.1) | 7(31.8) | Dominant | 0.543 |
|  |  | CC |  | 26(13.2) | 3(13.6) | Recessive | 1.000 |
|  |  |  | T | 265(67.3) | 31(70.5) | Allele | 0.688 |
|  |  |  | C | 129(32.7) | 13(29.5) |  |  |
| MSH4 | rs5745325 | GG |  | 189(94.5) | 21(95.5) | Codominant | 1.00 |
|  |  | GA |  | 11(5.5) | 1(4.5) | Dominant | 1.00 |
|  |  |  | G | 389(97.2) | 41(93.2) | Allele | 0.152 |
|  |  |  | A | 11(2.8) | 3(6.8) |  |  |
| MSH4 | rs5745549 | GG |  | 157(81.3) | 17(81) | Codominant | 0.832 |
|  |  | GA |  | 33(17.1) | 4(19) | Dominant | 1.00 |
|  |  | AA |  | 3(1.6) | 0 | Recessive | 1.00 |
|  |  |  | G | 347(89.8) | 38(90.5) | Allele | 1.00 |
|  |  |  | A | 39(10.2) | 4(9.5) |  |  |
| MSH5 | rs707938 | AA |  | 95(48.2) | 11(50) | Codominant | 0.168 |
|  |  | AG |  | 76(38.6) | 11(50) | Dominant | 0.874 |
|  |  | GG |  | 26(13.2) | 0 | Recessive | 0.084 |
|  |  |  | A | 266(67.5) | 33(75) | Allele | 0.312 |
|  |  |  | G | 128(32.5) | 11(25) |  |  |

Table S4. Association between PLT count and SNPS in AML

| Genes | SNPs | Genotype | Allele | Low PLT  *n* (%) | High PLT  *n* (%) | Model / allele | Uncorrected  *P* value |
| --- | --- | --- | --- | --- | --- | --- | --- |
| MLH1 | rs1799977 | AA |  | 45(93.8) | 156(90.2) | Codominant | 0.577 |
|  |  | AG |  | 3(6.2) | 17(9.8) | Dominant | 0.577 |
|  |  |  | A | 93(96.8) | 329(95.1) | Allele | 0.586 |
|  |  |  | G | 3(3.2) | 17(4.9) |  |  |
| MLH3 | rs175080 | GG |  | 36(75) | 100(57.8) | Codominant | 0.096 |
|  |  | GA |  | 11(22.9) | 67(38.7) | Dominant | **0.03** |
|  |  | AA |  | 1(2.1) | 6(3.5) | Recessive | 1.00 |
|  |  |  | G | 83(86.5) | 267(77.2) | Allele | 0.05 |
|  |  |  | A | 13(13.5) | 79(22.8) |  |  |
| PMS1 | rs5742933 | GG |  | 27(58.7) | 1 01(58.7) | Codominant | 0.996 |
|  |  | GC |  | 17(37) | 64(37.2) | Dominant | 0.998 |
|  |  | CC |  | 2(4.3) | 7(4.1) | Recessive | 1.00 |
|  |  |  | G | 71(77.2) | 266(77.3) | Allele | 0.975 |
|  |  |  | C | 21(22.8) | 78(22.7) |  |  |
| MSH2 | rs2303428 | TT |  | 25(53.2) | 80(46.8) | Codominant | 0.509 |
|  |  | TC |  | 18(38.3) | 66(38.6) | Dominant | 0.436 |
|  |  | CC |  | 4(8.5) | 25(14.6) | Recessive | 0.275 |
|  |  |  | T | 68(72.4) | 226(66.1) | Allele | 0.251 |
|  |  |  | C | 26(27.6) | 116(33.9) |  |  |
| MSH4 | rs5745325 | GG |  | 43(89.6) | 166(96) | Codominant | 0.104 |
|  |  | GA |  | 5(10.4) | 7(4) | Dominant | 0.104 |
|  |  |  | G | 91(94.8) | 339(97.9) | Allele | 0.145 |
|  |  |  | A | 5(5.2) | 7(2.1) |  |  |
| MSH4 | rs5745549 | GG |  | 41(87.2) | 132(79.5) | Codominant | 0.396 |
|  |  | GA |  | 6(12.8) | 31(18.7) | Dominant | 0.232 |
|  |  | AA |  | 0 | 3(1.8) | Recessive | 1.000 |
|  |  |  | G | 88(93.6) | 295(88.9) | Allele | 0.176 |
|  |  |  | A | 6(6.4) | 37(11.1) |  |  |
| MSH5 | rs707938 | AA |  | 21(44.7) | 84(49.1) | Codominant | 0.742 |
|  |  | AG |  | 19(40.4) | 68(39.8) | Dominant | 0.589 |
|  |  | GG |  | 7(14.9) | 19(11.1) | Recessive | 0.479 |
|  |  |  | A | 61(64.9) | 236(69.1) | Allele | 0.449 |
|  |  |  | G | 33(35.1) | 106(30.9) |  |  |

Table S5. Association between chemotherapy response in AML and SNPs.

| Genes | SNPs | Genotype | Allele | CR group  *n* (%) | Refractory group  *n* (%) | Model/allele | Uncorrected  *P* value |
| --- | --- | --- | --- | --- | --- | --- | --- |
| MLH1 | rs1799977 | AA |  | 131(89.7) | 71(93.4) | Codominant | 0.362 |
|  |  | AG |  | 15(10.3) | 5(6.6) | Dominant | 0.373 |
|  |  |  | A | 277(94.8) | 147(96.7) | Allele |  |
|  |  |  | G | 15(5.2) | 5(3.3) |  |  |
| **MLH3** | **rs175080** | GG |  | 80(54.8) | 57(75) | Codominant | **0.007** |
|  |  | GA |  | 62(42.5) | 16(21.1) | Dominant | **0.003** |
|  |  | AA |  | 4(2.7) | 3(3.9) | Recessive | 0.693 |
|  |  |  | G | 222(76.1) | 130(85.5) | Allele | **0.019** |
|  |  |  | A | 70(23.9) | 22(14.5) |  |  |
| PMS1 | rs5742933 | GG |  | 85(59) | 44(58.7) | Codominant | 0.996 |
|  |  | GC |  | 53(36.8) | 28(37.3) | Dominant | 0.959 |
|  |  | CC |  | 6(4.2) | 3(4.0) | Recessive | 1.00 |
|  |  |  | G | 223(77.4) | 116(77.3) | Allele | 0.982 |
|  |  |  | C | 65(22.6) | 34(22.7) |  |  |
| MSH2 | rs2303428 | TT |  | 69(48.3) | 37(38.7) | Codominant | 0.195 |
|  |  | TC |  | 59(41.2) | 25(32.9) | Dominant | 0.447 |
|  |  | CC |  | 15(10.5) | 14(18.4) | Recessive | 0.125 |
|  |  |  | T | 197(68.8) | 99(63.4) | Allele | 0.425 |
|  |  |  | C | 89(31.2) | 53(36.6) |  |  |
| MSH4 | rs5745325 | GG |  | 136(93.2) | 74(97.4) | Codominant | 0.228 |
|  |  | GA |  | 10(6.8) | 2(2.6) | Dominant | 0.228 |
|  |  |  | G | 282(96.5) | 150(98.6) | Allele | 0.234 |
|  |  |  | A | 10(3.5) | 2(1.4) |  |  |
| MSH4 | rs5745549 | GG |  | 110(78) | 64(87.7) | Codominant | 0.159 |
|  |  | GA |  | 28(19.9) | 9(12.3) | Dominant | 0.086 |
|  |  | AA |  | 3(2.1) | 0 | Recessive | 0.553 |
|  |  |  | G | 248(87.9) | 137(93.8) | Allele | 0.055 |
|  |  |  | A | 34(12.1) | 9(6.2) |  |  |
| MSH5 | rs707938 | AA |  | 70(48.6) | 36(48) | Codominant | 0.897 |
|  |  | AG |  | 56(38.9) | 31(41.3) | Dominant | 0.932 |
|  |  | GG |  | 18(12.5) | 8(10.7) | Recessive | 0.691 |
|  |  |  | A | 196(68.1) | 103(68.6) | Allele | 0.896 |
|  |  |  | G | 92(31.9) | 47(31.4) |  |  |

Table S6. Association between MRD status in AML and SNPs

| Genes | SNPs | Genotype | Allele | MRD positive  *n* (%) | MRD  negative  *n*(%) | Model/allele | Uncorrected *P* value |
| --- | --- | --- | --- | --- | --- | --- | --- |
| MLH1 | rs1799977 | AA |  | 108(93.1) | 84(87.5) | Codominant | 0.165 |
|  |  | AG |  | 8(6.9) | 12(12.5) | Dominant | 0.165 |
|  |  |  | A | 224(96.5) | 180(93.75) | Allele | 0.176 |
|  |  |  | G | 8(3.5) | 12(6.25) |  |  |
| MLH3 | rs175080 | GG |  | 77(66.4) | 53(55.2) | Codominant | **0.045** |
|  |  | GA |  | 38(32.7) | 37(38.5) | Dominant | **0.096** |
|  |  | AA |  | 1(0.9) | 6(6.3) | Recessive | **0.048** |
|  |  |  | G | 192(82.8) | 143(74.5) | Allele | **0.037** |
|  |  |  | A | 40(17.2) | 49(25.5) |  |  |
| PMS1 | rs5742933 | GG |  | 69(60.5) | 59(61.5) | Codominant | 0.99 |
|  |  | GC |  | 40(35.1) | 33(34.4) | Dominant | 0.89 |
|  |  | CC |  | 5(4.4) | 4(4.2) | Recessive | 1.00 |
|  |  |  | G | 178(78.1) | 151(78.6) | Allele | 0.887 |
|  |  |  | C | 50(21.9) | 41(21.4) |  |  |
| MSH2 | rs2303428 | TT |  | 58(50) | 44(47.3) | Codominant | 0.77 |
|  |  | TC |  | 42(36.2) | 38(40.9) | Dominant | 0.699 |
|  |  | CC |  | 16(13.8) | 11(11.8) | Recessive | 0.674 |
|  |  |  | T | 158(68.2) | 126(67.7) | Allele | 0.937 |
|  |  |  | C | 74(31.8) | 60(32.3) |  |  |
| MSH4 | rs5745325 | GG |  | 111(95.7) | 90(93.8) | Codominant | 0.551 |
|  |  | GA |  | 5(4.3) | 6(6.2) | Dominant | 0.551 |
|  |  |  | G | 227(97.8) | 186（96.8） | Allele | 0.555 |
|  |  |  | A | 5(2.2) | 6(13.2) |  |  |
| MSH4 | rs5745549 | GG |  | 94(84.7) | 72(76.6) | Codominant | 0.088 |
|  |  | GA |  | 15(13.5) | 22(23.4) | Dominant | 0.141 |
|  |  | AA |  | 2(1.8) | 0 | Recessive | 0.501 |
|  |  |  | G | 203(91.5) | 166(88.3) | Allele | 0.29 |
|  |  |  | A | 19(8.5) | 22(11.7) |  |  |
| MSH5 | rs707938 | AA |  | 53(46.9) | 49(51) | Codominant | 0.647 |
|  |  | AG |  | 45(39.8) | 38(39.6) | Dominant | 0.551 |
|  |  | GG |  | 15(13.3) | 9(9.4) | Recessive | 0.378 |
|  |  |  | A | 151(66.8) | 136(70.8) | Allele | 0.377 |
|  |  |  | G | 75(36.2) | 56(29.2) |  |  |

Table S7 Multivariate Cox regression analysis of *MSH2* rs2303428 recessive model and OS

| Characteristics | Total(*n*) | HR (95% CI) | *P* value |
| --- | --- | --- | --- |
| Sex | Female(n=105) | reference |  |
|  | Male(n=112) | 1.21(0.89-1.68) | 0.223 |
| Age (Years) | ﹤60(*n*=160) | reference |  |
|  | ≥60(*n*=57) | 2.60(1.82-3.71) | ＜0.001 |
| Risk group | Favorable(*n*=64) | reference |  |
|  | Intermediate/adverse(*n*=153) | 1.17(0.82-1.69) | 0.384 |
| WBC (×10^9^/L) | ﹤100(*n*=195) | reference |  |
|  | ≥100(*n*=22) | 2.66(1.61-4.37) | ＜0.001 |
| Rs2303428 |  |  |  |
| Recessive | TT/TC(n=189) | reference |  |
|  | CC(n=28) | 1.66(1.06-2.58) | 0.027 |

Table S8 Multivariate Cox regression analysis of *MSH4* rs5745549 dominant model and PFS

| Characteristics | Total(*n*) | HR (95% CI) | *P* value |
| --- | --- | --- | --- |
| Sex | Female(n=83) | reference |  |
|  | Male(n=91) | 1.05(0.74-1.48) | 0.766 |
| Age (Years) | ﹤60(*n*=127) | reference |  |
|  | ≥60(*n*=47) | 1.24(0.85-1.81) | 0.24 |
| Risk group | Favorable(*n*=52) | reference |  |
|  | Intermediate/adverse(*n*=122) | 1.24(0.82-1.87) | 0.29 |
| WBC (×10^9^/L) | ﹤100(*n*=154) | reference |  |
|  | ≥100(*n*=20) | 2.28(1.33-3.91) | 0.003 |
| Rs5745549 |  |  |  |
| Dominant | GG(*n*=140) | reference |  |
|  | GA/AA(*n*=34) | 0.68(0.42-1.06) | 0.091 |

Table S9 Multivariate Cox regression analysis of *PMS1* rs5742933 recessive model and PFS

| Characteristics | Total(*n*) | HR (95% CI) | *P* value |
| --- | --- | --- | --- |
| Sex | Female(n=86) | reference |  |
|  | Male(n=94) | 1.01(0.71-1.41) | 0.97 |
| Age (Years) | ﹤60(128) | reference |  |
|  | ≥60(*n*=50) | 1.24(0.85-1.81) | 0.25 |
| Risk group | Favorable(*n*=55) | reference |  |
|  | Intermediate/adverse(*n*=123) | 1.26(0.86-1.86) | 0.23 |
| WBC (×10^9^/L) | ﹤100(*n*=159) | reference |  |
|  | ≥100(*n*=19) | 2.34(1.37-3.99) | 0.002 |
| Rs5742933 |  |  |  |
| Recessive | GG/GC(*n*=170) | reference |  |
|  | CC(*n*=8) | 3.03(1.38-6.63) | 0.006 |

| Genes | SNPs | Allele | AML  n(%) | East Asian Controls  (%) | Uncorrected *P* value |
| --- | --- | --- | --- | --- | --- |
| MLH1 | rs1799977 | A | 424(95.5) | 97.32 |  |
|  |  | G | 20(4.5) | 2.68 | 0.721 |
| MLH3 | rs175080 | G | 352(79.3) | 83.3 |  |
|  |  | A | 92(20.7) | 16.7 | 0.471 |
| PMS1 | rs5742933 | G | 339(77.4) | 100 |  |
|  |  | C | 99(22.6) | 0 | **＜0.001** |
| MSH2 | rs2303428 | T | 296(67.5) | 70.7 |  |
|  |  | C | 142(32.5) | 29.3 | 0.573 |
| MSH4 | rs5745325 | G | 432(97.3) | 97.3 |  |
|  |  | A | 12(2.7) | 2.7 | 1.00 |
| MSH4 | rs5745549 | G | 385(89.95) | 99.22 |  |
|  |  | A | 43(10.05) | 0.78 | **0.005** |
| MSH5 | rs707938 | A | 299(68.3) | 62.9 |  |
|  |  | G | 139(31.7) | 37.1 | 0.457 |

Table S10 Association between gene frequencies status in AML and East Asian Controls
